# Supplementary material for: MorphoGraphX: A platform for quantifying morphogenesis in 4D
Source: eLife. 2015 May 6;4:e05864. doi: 10.7554/eLife.05864 (PMC4421794; doi:10.7554/eLife.05864)
Supplement: Supplementary file 1. — MorphoGraphX User Manual. The MorphoGraphX user manual is written in a tutorial style, and the accompanying data sets are available for download on the MorphoGraphX website (www.MorphoGraphX.org). Installation instructions for MorphoGraphX and troubleshooting tips are in Section 16 towards the end of the manual. DOI: http://dx.doi.org/10.7554/eLife.05864.022 [file elife05864s001.pdf]

3D Image processing with MorphoGraphX
User manual

www.MorphoGraphX.org

This manual will introduce you step by step to 3D image processing and analysis software in MorphoGraphX. Example confocal stacks from the tutorial can be downloaded from www.MorphoGraphX.org.

The manual covers the software basics:

- MorphoGraphX Installation.
- How to get good quality confocal data for segmentation.
- Loading and viewing confocal image data.
- Extracting the cell outline on a curved surface (2½D segmentation).
- Analysis of growth from time-lapse data and quantification of fluorescence.
- Exporting results into other formats.
- 3D segmentation.

If you are only interested in using the 3D segmentation, we recommend nevertheless to go through the entire tutorial since the procedures are very similar for curved surface (2½D) and 3D, with the exception of chapter 13 (Principal Directions of Growth).
New functions are constantly added to the software. For a more detailed description of new or advanced function, please refer to the internal documentation or visit www.MorphoGraphX.org.

Table of Contents

User Guide....4
- 1. Introduction to MorphoGraphX....4
  - Some definitions....4
  - User interface in MorphoGraphX....4
  - Main menu, toolbars and "Main" tab....5
  - "View" tab....6
  - "Process" tab....7
- 2. Data collection....7
- 3. Loading samples (stack or mesh)....9
  - Troubleshooting....10
- 4. Extract the biological object shape....11
  - Troubleshooting....12
- 5. Experiment with Processes....13
- 6. Create a 2½D image....14
- 7. Segment the cells....14
  - Manual seeding and segmentation....15
  - Automatic seeding and segmentation....16
  - Troubleshooting....17
- 8. Refine the subdivision....17
- 9. Quantify Cell Area....18
- 10. Quantify Fluorescence....19
- 11. Parent labeling....20
  - Troubleshooting....22

12. Heat map of growth and cell proliferation....22
- 13. Principal directions of growth (PDGs)....24
- 14. 3D segmentation....28
  - Troubleshooting....29

15. Create your own workflow (tasks)....29
Reference....30
16. Installation....30
After installation – enabling the ALT key....30
Laptop use....30
Troubleshooting....31
Cuda 6.5....31
17. Writing custom processes (plugins)....32
18. Compiling MorphoGraphX from source....33
Linux....33
Windows....33
Mac....34
19. File formats....34
20. Command line options....34

User Guide

1. Introduction to MorphoGraphX

MorphoGraphX is an open source platform for the visualization and processing of 3D image data. MorphoGraphX can manipulate 3D image stacks and use them to extract curved surfaces (2½D) from them or segment data in full 3D.

Some definitions

- **Stack.** A *stack* corresponds to a 3D image of a biological sample. There can be 2 *stores* associated with each stack, corresponding for example to the original 3D image and the same data after image processing. The size of a stack is defined in *voxels*, which are the equivalent of pixels for 3D images.

- **Store.** There are 2 stores in each stack. Usually the original sample is loaded into the *main store.* Once the original stack has been modified by a function, the result (e.g. the 3D image after blurring) is stored separately in the *work store*, without over-writing the original image in the main store. Each store has a separate color, brightness, transparency, etc.

- **Mesh**. A *mesh* is a triangulated curved surface. Typically the mesh is extracted from a stack after some image processing operations are performed on it. Currently MorphoGraphX can handle 2 stacks (each with a main and work store) and 2 associated meshes in a single session.

- **Process.** Most operations in MorphoGraphX are performed by processes. There are 3 kinds of processes, “*Mesh*”, “*Stack*”, and “*Global*”. They are named after what they can modify. Processes can be organized by users into custom pipelines called *tasks*.

User interface in MorphoGraphX

Start a MorphoGraphX session with the example data loaded by clicking on the “example_session.mgxv” file in the “Kierzkowski_2012” folder.

Main menu, toolbars and “Main” tab.

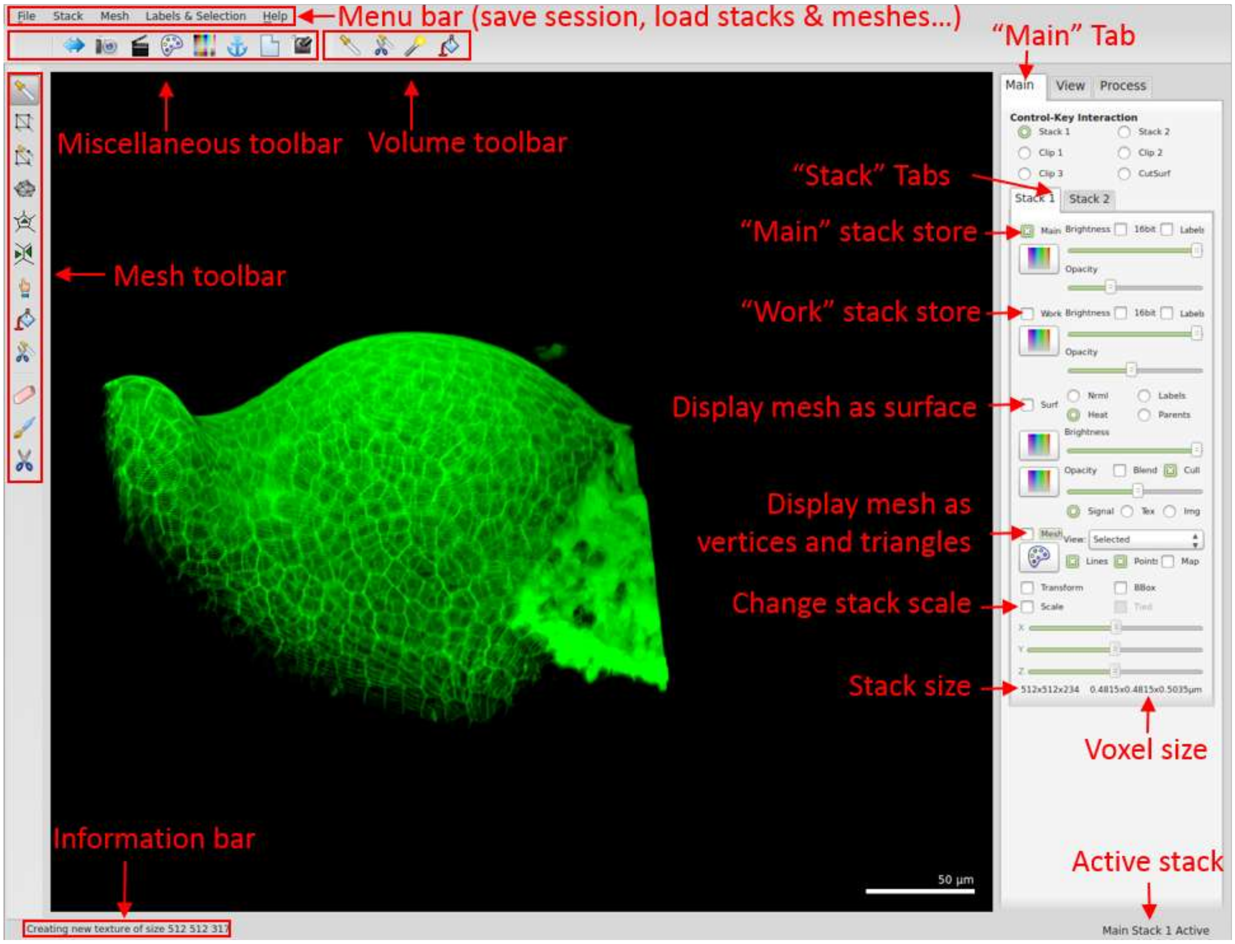

Tools from the **toolbars** will work only if the “Alt”-key is pressed on. Some of the tools are enabled under specific conditions, which will be described in the information bar if you try to use them. You can drag and drop the toolbars around to change their position in the MorphoGraphX window.

Since two different stacks (Stack1 and Stack2) and associated meshes can be displayed simultaneously, you have to specify which one is being processed (**Active stack**). Select the active stack and mesh by switching between the **Stack1** and **Stack2** tabs.

The input for stack operations (3D filters and the like) is chosen by checking either the **Main** or **Work store**. The output (modified image) is always stored in the Work store.

“View” tab

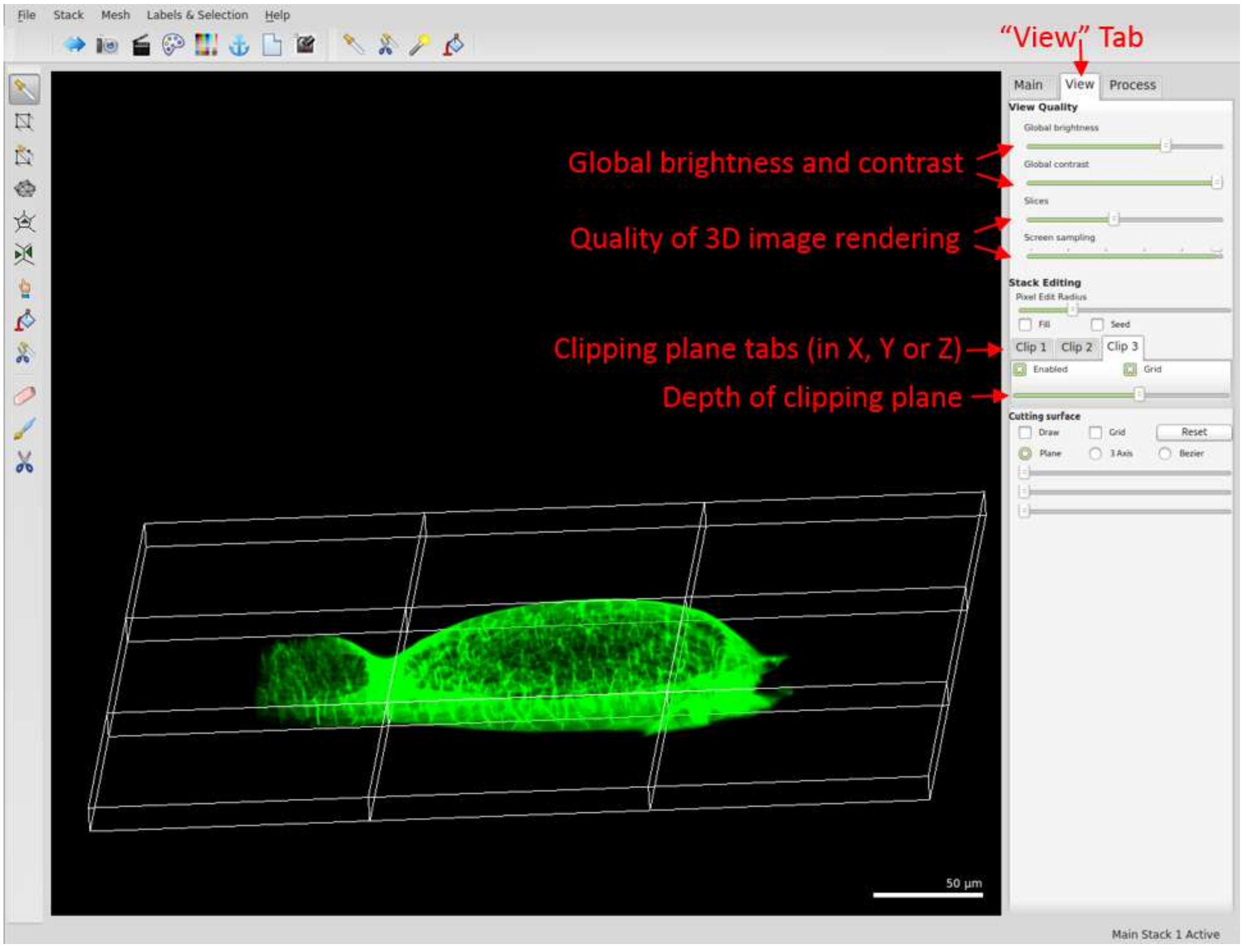

Global brightness and contrast can be tuned in the **View tab**, as opposed to the brightness and contrast of individual stacks and meshes that are controlled from the “Main” tab. Depending on the speed of your machine, 3D rendering can run fast or slow. You can alter the quality of 3D images display using the **Slices** and **Screen sampling** slides to make 3D rendering faster when the stacks are rotated.

“Process” tab

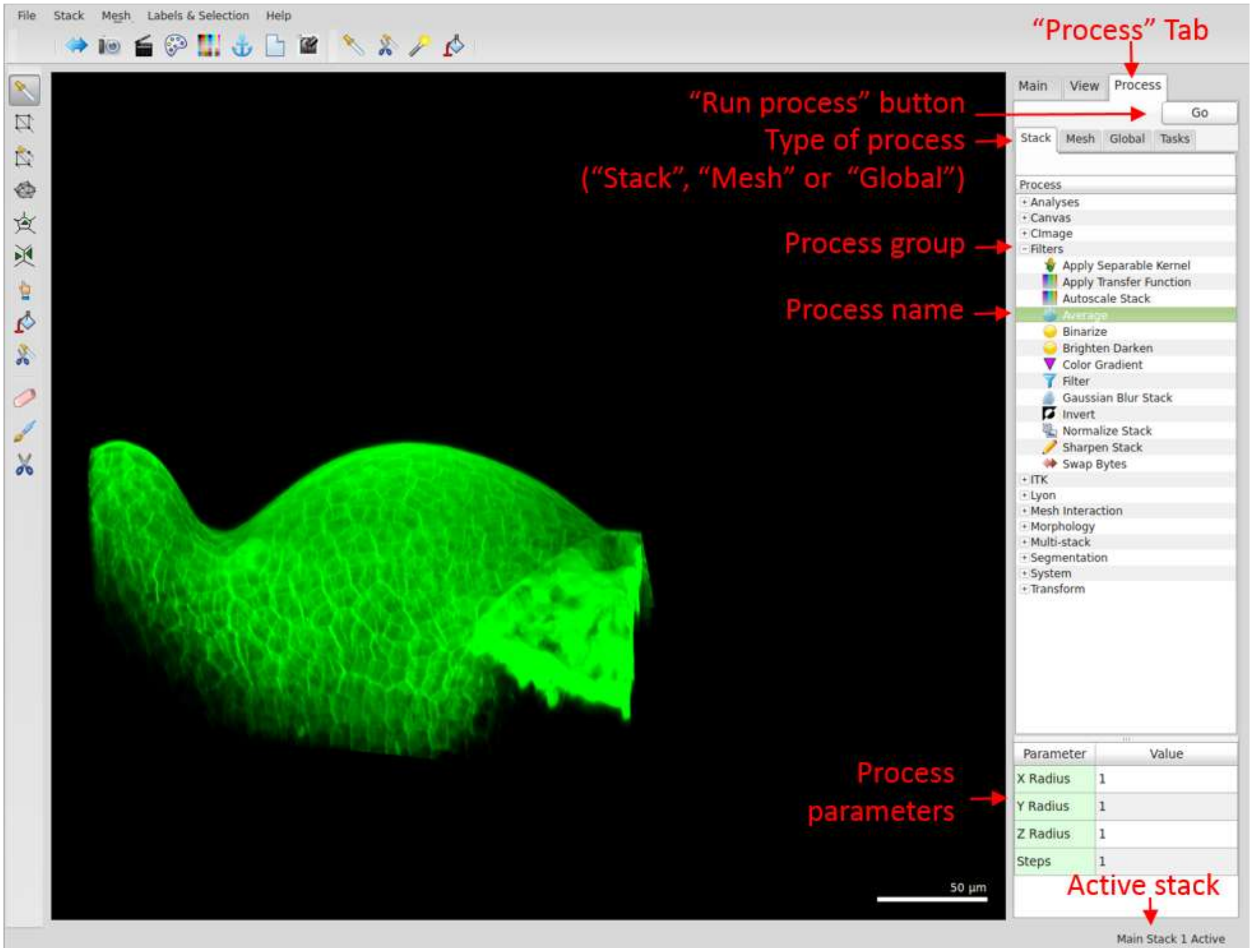

Processes are bundled in **groups**, that you can unfold by clicking on the “+” icon next to the group name. To run a **process**, press the “Go” button or double click on the process name. While running a process, make sure that the right stack number (1 or 2) and store (“Main” or “Work”) is active.

2. Data collection

MorphoGraphX has mostly been used to process 3D confocal image stacks. Data collection for use in MorphoGraphX can be a little different than for other uses. In most cases, people optimize data collection so that individual slices look good to the human eye. This is not always the best for 3D data analysis and visualization. The following are some tips to help get the most out of the images:

- **16 bits images**. If possible collect 16 bits per channel, instead of 8 bits. Although the pictures may look no different, the 16 bits images will have higher dynamic range, and it

will be easier to extract features in darker areas of the image. If the microscope only supports 12 bits, that will still be better than 8 bits.

- **Cubic voxels.** Use a small z-step. Try to make the voxels close to cubic for best results. For example, if the XY resolution is 0.5 µm, then use 0.5 or 1 µm for the z step (0.5 is better).
- **Averaging.** There is always a trade between resolution and how much exposure the sample can tolerate, as well as how much time you have. Frame averaging is used to get better individual slices, but requires multiple scans per slice. For 3D image processing it is generally better to have more "real" slices. For example, if you have 0.5 µm for XY pixel size, and are using a z-step of 2 µm with 4x frame averaging, it would be better to turn the frame averaging to 1 and set the z-step to 0.5 µm. Both should have the same exposure time, but of course without frame averaging you get a 4x larger data file.
- **Image saturation.** It is best to have the images just below the point where they are saturated. The controlling software of confocal microscopes usually has a mode to show saturated pixels. Turn the gain up until you just start to see some saturated pixels. Of course if you want to compare fluorescence data between samples to make a quantification of fluorescence levels, it is necessary to not saturate the channels you want to quantify. For wall stains where you do not need to quantify, it can help to over-saturate in order to get a good contrast between the inside and the outline of the cell.
- **Wall Stain.** To get a nice segmentation you need a method to stain the cell walls or the plasma membrane. This can be done with propidium iodide (PI) or a marker line. With the correct detection range PI can be reliably separated from both GFP and YFP using the same excitation wavelength. Since most confocal microscopes can capture different light frequencies to different channels simultaneously, you can collect PI wall stain signal, and GFP fluorescence marker signal in the same amount of time/exposure. MorphoGraphX will use the PI wall stain for surface extraction and segmentation, and then the GFP channel for gene expression analysis. You will have to experiment a bit to get the best results for your samples.
- **Start in the right place.** Be sure to start data collection a few microns above the sample. Many people are tempted to start collection when they see the first cells. This will leave a flat spot at the top of the sample. Also be careful not to miss the bottoms of cells if you are doing 3D segmentation, as you can only quantify cells which are complete.
- **Time-lapse imaging.** If you want to quantify growth or changes in signal intensity over time, the same sample may have to be imaged multiple times, up to several days. Repeated exposure to a light source (e.g. confocal laser) results in considerable stress for biological samples and might interfere with the results. The time intervals between image acquisitions should be long enough to allow the sample to recover. On the other hand, the sample should not grow too much in between images, otherwise it can become too difficult to identify the cells between successive time points. As a rule of thumbs, the cells should not divide more than twice during the time intervals.
- **Use Fiji to get TIF files.** Most microscope formats are proprietary. The Loci bio-formats group has reverse engineered the formats of the most popular microscopes and made plugins for ImageJ. We recommend Fiji, which is a distribution of ImageJ that contains

these, along with many other useful plugins. After opening a stack in Fiji, split it into separate windows for each channel, as MorphoGraphX currently requires there be only one channel per file. From Fiji, save each channel as a multilayer TIF file.

- **If voxel sizes are wrong.** The TIF format has no standard way to specify the voxel size in Z. ImageJ writes this its own way, and MorphoGraphX is programmed to read it. If this doesn't work for some reason then MorphoGraphX allows you to change the voxel size after you load the image (see chapter 3b). If you need to do this, you can get the voxel sizes using the microscope software or from the metadata (text file) in ImageJ.

3. Loading samples (stack or mesh)

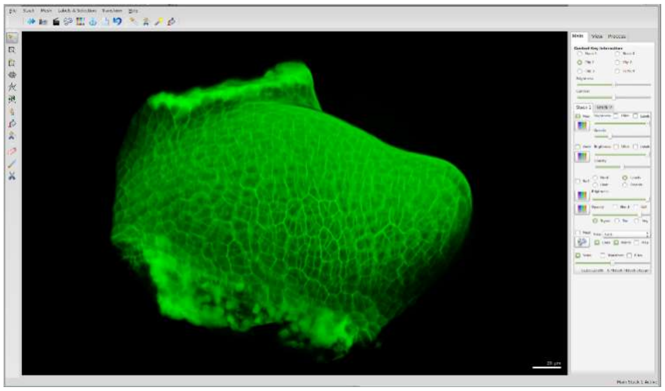

The first step is to load your sample into MorphoGraphX:

- **Start MorphoGraphX.** If there is a green MGX icon on your desktop, double click the icon. Another way to start MorphoGraphX is to open an existing session by double-clicking on a “.mgxv” file. You can even create an empty “.mgxv” file and open it. After MorphoGraphX starts you will see the main window and a text window (called terminal) underneath. Do not close the terminal, if you do, MorphoGraphX will shut down. Informational messages from processing operations will be displayed in the terminal.
- **Load a stack.** Drag and drop a TIF image stack onto the main window. This will load your sample into the main store of Stack1. Drag and drop the file with the Alt-key pressed to load the sample into Stack2. You can also load samples with the Stack menu (for example under “Stack/Stack1/Main/Open”).
- **Importing file series.** It is also possible to import image series with the “Import series” option in Stack menu. In this case, you will need to specify the Z-step yourself.
- **Loading a pre-existing mesh.** Meshes are usually extracted from stacks, but they can also be loaded separately from the stacks. Load meshes the same way as stacks, either by dragging and dropping the files into the main MorphoGraphX window, or by using the Mesh menu (e.g. “Mesh/Mesh1/Load”).
- **Experiment with the visualization tools.** MorphoGraphX uses QGLViewer to handle the display of samples. The left mouse button allows you to rotate the sample, the right button (or left button + Shift-key) translates, and the wheel is used to zoom. Mouse and

keyboard codes are listed under “About QGLViewer” in the Help menu. There are many visualization tools in MorphoGraphX. We recommend to load two different samples in Stack1 and Stack2 and take some time to experiment with:

1. **Rotation-translation**. Left click on the display area and moving the mouse rotates both stacks (that is, changes the camera view angle), while the right click translates the point of view. You can check the camera movements relative to the coordinate system (X, Y, Z) by pressing the A-key. Double left-click to rotate the camera to the nearest view aligned with one of the axes(X, Y or Z). To move each stack independently, press the Ctrl-key. Specify which stack should be displaced using the check boxes in “Control-Key interaction” within the Main tab. Reset all the rotations and translations clicking on the icon on the 'miscellaneous' toolbar. Press the A-key again to hide the coordinate system.

2. **Transfer functions**. In the Main Tab, click on the icon to display the color scale, or transfer function, of each stack. A pop-up window will appear. Use the “Predefined color maps” menu to change the type of transfer function. Double-click on the histogram to add a marker (black triangle) and displace the markers to modify the color map. Right-click on the histogram to reverse the transfer function.

3. **Clipping planes**. In the “View” tab, select one of the clipping planes in X, Y or Z in the Clip1, Clip2 and Clip3 tabs. To display the boundaries of the clipping plane, check the “Grid” checkbox on. Modify the depth of clipping plane with the slider. To display only the part of the stack inside the clipping plane, check “Enabled” on. You can move the clipping planes around the stack by holding the Crtl-key. Note that the correct radio button for the Control-Key-Interaction must be set in the View tab. Reset the clipping planes rotation with the button. To get a cross section of the sample, align the camera angle with one of the axes (e.g. Z) and enable the clipping plane perpendicular to that axis (e.g. Clip3) to obtain a cross section. Select the Control-key interaction for this plane. Slice throughout the sample using the middle mouse button while pressing the Crtl-key. You can visualize more than one clipping plane at the same time.

4. **Slices**. Under “View Quality” in the View tab, use the “Slices” slider to modify the number of stack slices displayed. Try to rotate the stack while the slice number is either minimal or maximal and notice if it makes a difference in the rendering speed.

- **Save the MorphoGraphX session.** You can save the session with all the parameters used and the path of the data loaded. In the File menu, select “save” or “save as”. This will create a MorphoGraphX session (.mgxv file) that you can start next time. The data (stacks and meshes) will then be automatically loaded.

Troubleshooting

- Symptom: “**The stack is loaded, but not visible**”.

  Try the following solutions in order:

  - *The stack or mesh can be out of the field of view.* Use the to reset the view.

- *The stack color depth might be 8 or 12 bits instead of 16 bits*. By default the stacks are supposed to be 16 bits and the color-mapping is optimized for this color depth. Check the transfer function by clicking on 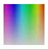 in the main store of the stack you just loaded. In the “Transfer function editor” window, click on “Auto Adjust”. If the data is 8 or 12 bits, only part of the histogram will be filled (1/16 or the full range in case of 12 bits data, 1/256 in case of 8 bits). Although it is possible to run all the stack processes on non-16 bits images, we recommend you to re-scale the data to 16 bits processing it. Use “Process/Stack/Filters/Brighten Darken” with an amount of 16 to convert 12 bits images, or 256 for 8 bits images. Next run “Process/Stack/Multi-stack/Copy work to Main stack”. Reset the transfer function of the main stack in the “Transfer function editor”. Now the stack should look fine and you can use it for further processing. Save it using the Stack menu (you can over-write the original stack or save separately the 16 bits version).
- *The stack voxel size might be wrong*. In the Main tab, check the voxel size (see chapter 1b). If it does not correspond to the pixel size and the z-steps used during acquisition, resize the voxels using “Process/Stack/Canvas/Change Voxel Size”. If the size is already correct in some of the directions (X, Y or Z), enter a value of 0 for these and their size will not be changed.
- *The store into which you loaded your data might not be active*. Check the 'main' and 'work' checkbox in Main tab.

4. Extract the biological object shape

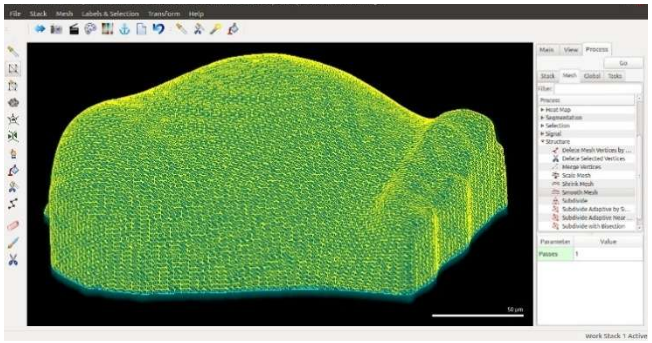

The first step in the segmentation process is to extract the global shape of your sample. This is performed by first blurring the sample slightly to reduce noise, and then using filters to convert the object to a solid shape. The surface of the object is then extracted from this shape as a triangular mesh. To create a mesh, perform the following:

- **Load the stack.** Make sure that the PI stack is loaded into Stack1-Main and Main Stack 1 is active.
- **Blur the stack.** Run “Process/Stack/Filters/Gaussian Blur Stack” with values of 0.3, or “Process/Stack/Filters/Average”. The blurred image is stored in Work stack, and the active stack is automatically changed to Work Stack 1.
- **Edge detect.** Use “Process/Stack/Morphology/Edge detect”. This will create a solid shape representing the global shape of your object. Tip: for optimal visualization of this solid shape, turn the opacity of the work stack to the maximum. At this point it is also possible to use the “Pixel Edit” tool with Alt-key to erase parts of the stack that you do not need. Note that this operation only works on the work stack.
- **Extract the surface.** Run “Process/Mesh/Creation/ Marching Cubes Surface”, with a cube size of 5 µm. As a general rule, the cube size should be roughly as large as the cells if you want to extract only the general organ shape, and several times smaller if you want to capture the curvature of individual cells.
- **Trim off the bottom.** In the Main tab, ensure that the Mesh checkbox, the “Lines” checkbox and the “Points” checkbox are selected, and “View” option is set to “All”. This will enable the visualization of the mesh. Click the “Select points in mesh” tool on the left and hold the Alt-key to select the bottom vertices of the apex. They should turn red. Hit the delete key to remove them. To make this easier, it is nice to have the apex in a horizontal position. You can do this by left-double clicking on it. Try to delete the bottom cleanly.
- **Smooth the mesh.** Run “Process/Mesh/Structure/Smooth Mesh” several times. You should now have a mesh following the global shape of the sample. Check how accurate it is with the clipping planes. Be sure to turn off the work store, and turn the main store back on when you do this. As the clipping planes are made thinner, the image will get darker, you can increase the opacity to counteract this. You want your mesh to resemble the shape of the original data. If the mesh is far away from the surface (does not align tightly with the shape of your stack), increase the threshold for the edge detect. For the sample data try 25,000.

Troubleshooting

- Symptom: “**The Edge Detect results in a very strange shape**”. If the stack is loaded upside-down, the Edge Detect will extract the shape of the sample’s bottom. Check if the stack is loaded in the correct orientation by pressing the “A” key and the “Reset view” button. This will show you the orientation of the X, Y and Z axis. If the arrow of the Z axis is pointing downward, run “Process/Stack ->Canvas->Reverse Axes”, with parameters: (X “No”, Y “No”, Z “Yes”). You can then over-write your stack for further use. Press the “A” key again to make the axis arrows disappear.
- Symptom: “**The mesh is full of holes**”. Try first to run the whole procedure again with a lower threshold for the Edge Detect. If this does not help, use “Process/Stack/Morphology/Fill Holes” just after running the Edge Detect.

- Symptom: **“There are spikes on the sample shape after Edge Detect”**. The threshold for edge detection is probably too low.

5. Experiment with Processes

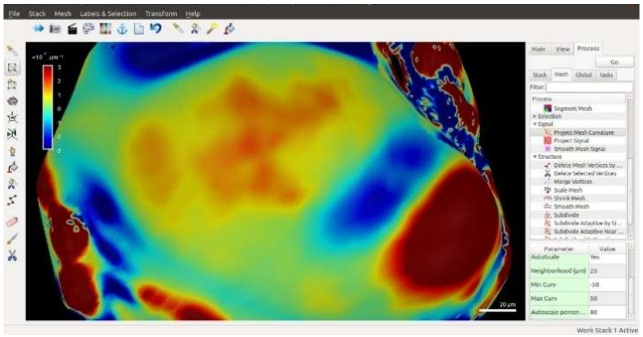

All data sets present different features and there is no universal recipe to process them in the best way. We recommend to experiment with processes, in order to adapt them to your data. The parameters we recommend for the sample data will not necessarily be optimal for your data. NB: remember to check which stack is currently active, so that you know if the process runs on the original data (Main store) or on an image already modified by the last process (Work store).

- **in “Process/Stack/Filters”:**
    - **Gaussian Blur Stack**. Try different values for the Sigma parameter (i.e. the radius of Gaussian blur). Compare the result (in Work store) and the original data (in Main store) using the clipping planes to get only a cross section. Larger radius should be more efficient at reducing the image noise, but also results in a more blurry image.
    - **Normalize Stack**. This will increase the contrast in the image, enhancing both the speckle noise and low signal areas. As above, use the clipping planes to observe the effect of normalization on the stack. You can perform a Gaussian blur before normalization to get the best results.
    - **Invert**. Transforms low signal into high signal and vice-versa. This can be useful if you want to segment features which are dark in the original image.
- **in “Process/Stack/Morphology”:** perform all the following operations with a clipping plane enabled to observe the effect on a cross section
    - **Dilate**.
    - **Erode**.
    - **Close**. This is just Dilation followed by Erosion.
- **in “Process/Mesh/Signal”:**
    - **Project Mesh Curvature**. Project the Gaussian curvature. For best results, use change the transfer function to Jet. Experiment with different neighborhood and Autoscale values.

6. Create a 2½D image

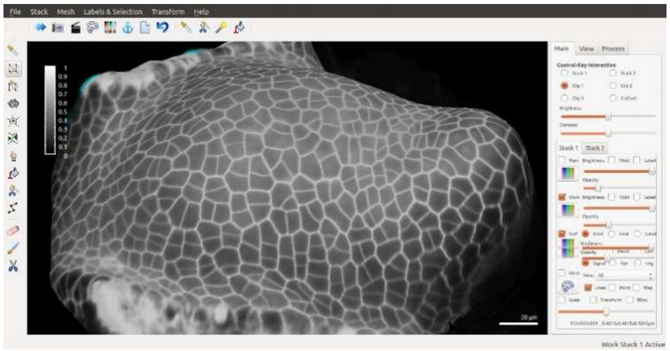

Many samples have too much curvature to simply segment them in 2D. This is one of the features of MorphoGraphX distinguishing it from other software. It was designed to work on curved “2½D” images. To create this image perform the following:

- **Subdivide the mesh.** Run “Process/Mesh/Structure/Subdivide”, then run Smooth Mesh process.
- **Project the signal onto the mesh.** Ensure that the Surface checkbox is selected, and deselect the Mesh checkbox. Then run the “Process/Mesh/Signal/Project Signal”. The stack Main store must be selected and the Work store deselected, so that the original data is projected, not the processed stack. At this point you should see the outlines of the cells. Notice the parameters: Minimum and Maximum Distance. These distances are in the negative direction of the surface normal and tell MorphoGraphX which part of the signal to project onto the surface. For better visualization of the mesh, you can turn down the opacity of the stack with the slider in the Main tab.
- **Save your work.** There is no Undo operation in MorphoGraphX, so it is important to save your work frequently. In the Mesh menu, choose Mesh1 and Save. Give your mesh a meaningful name such as “Coarse Mesh T0”, for example.
- **Visualize projected data.** If you would like to see exactly what data was projected onto the mesh, run “Process/Stack/Mesh Interaction/Annihilate”. Make sure the distances are the same as those you used for the projection. If you want to keep this stack you can use the stack menu option Stack1-Save. You have now created a “2½D” image of the surface layer of cells in the meristem. Have a look to see how it compares with the original sample data. You can adjust the mesh and stack opacity to see both the labels and the signal at the same time. If you turn on the mesh visualization to Cells, you will see the wireframe outlines of the cells.

7. Segment the cells

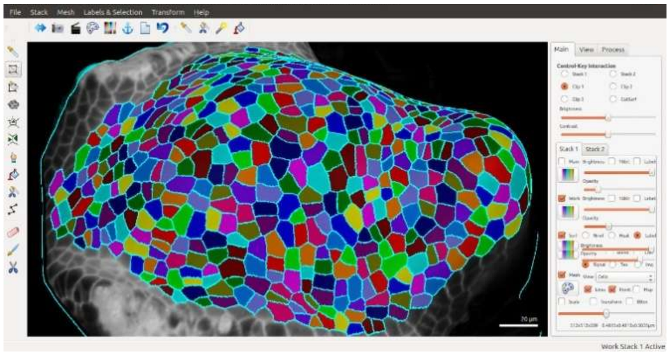

It is now time to segment the image into cells. The segmentation is done by propagating label seeds on the mesh surface using the watershed algorithm. Label seeds can be placed either manually or automatically. While the automatic seeding saves a lot of time, we recommend to get familiar with the manual seeding first, as it helps understand how to use the automatic procedure to its full capacity. Manual corrections are also often needed in areas where the signal is less clear and leads to errors in the automatic segmentation.

Manual seeding and segmentation

MorphoGraphX uses manual seeding and the watershed algorithm for this process:

- **Blur the cell outlines.** Run “Process/Mesh/Signal/Gaussian Blur”, or “Smooth Mesh Signal”. This will close potential gaps in the signal at the cell borders. For the Gaussian blur, use a radius close to the width of the cell border (here, 1 µm or below). Note that too much blurring can result in faulty segmentation.
- **Seed the cells.** Select the “Add new seed” tool . Ensure that the Surface and Labels checkboxes are selected. With Alt pressed, click on each cell to add a seed. If you click and hold you can draw on the surface with the seed. This can help to direct the segmentation, or to label two cells with the same color. When finished, draw a line around the entire area with a single seed. You can hold shift to continue with the current seed, or select the “Add current seed” tool .
- **Propagate the labels with watershed.** Once all the cells of interest are seeded, run “Process/Mesh/Segmentation/Watershed Segmentation”. Probably you will have segmentation errors. They are easy to see by turning the Mesh visualization on and selecting Cells in the dropdown beside. To fix them, first clear the current label in the top left by clicking on the icon (see chapter 1b, “Miscellaneous toolbar”). Then use the “Fill label” tool from the Mesh toolbar (*not* the Volume toolbar!) to clear the incorrect cells. You can then re-seed the empty cells using “Add new seed” and re-run the watershed. If you first select a label with the “Pick label” tool from the Mesh toolbar,

you can then color cells with that label by using “Fill label” 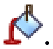. At this point it is very important to save your data.

Automatic seeding and segmentation

The automatic seeding and segmentation consist of several sub-processes which are described below for better understanding of the parameters which the user is supposed to set.

Please note: you can perform it as one process or as a series of separate processes. We suggest to try the latter option at the beginning because it will allow you to optimize the parameters for your sample and therefore make the procedure more effective.

- **Blur the cells.** Run “Process/Mesh/Signal/Gaussian Blur”. Estimate the radius of the smallest cells in your sample (in micrometers) and put it in the “radius” window. Try several values at the first attempt, as over- or under- estimation can result in incorrect segmentation. For the sample data, try 2 µm.

- **Seed the cells automatically.** Run “Process/Mesh/Segmentation/Auto-Seeding”. A seed will be put at local minima of signal (dark regions) within a given radius. Set the same cell radius as in the previous process, i.e. the radius of the smallest cells (here, 2 µm). If you use a larger radius the small cells might be under-segmented (fused).

- **Re-project the signal onto the mesh.** Run “Process/Mesh/Signal/Project Signal” with the same Min and Max distance parameters as originally used (see chapter 6).

- **Blur the cell outlines.** Run “Mesh/Signal/Gaussian Blur”, with a radius roughly equal to half the cell outline width (try 1 µm or below).

- **Segment the cells.** Run the watershed (“Process/Mesh/Segmentation/Watershed Segmentation”) as in manual segmentation. Probably you will observe that in some cases one cell has been seeded several times (i.e. is over-segmented). Merge the over-segmented cells based on signal using “Process/Mesh/Segmentation/Combine Labels” (described below).

- **Normalize mesh signal (optional).** This process can enhance the color contrast between the inside of the cell and its borders, which is important for the next step (merging cells). However, if the signal is strong and clear, it is not necessary to run this process. To try it, run “Process/Mesh/Signal/Normalize Signal”. This time indicate a radius slightly higher than the average radius of your cells (in the meristem sample data, 5 µm), not the radius of the smallest ones.

- **Merge the over-segmented cells.** Run **“**Process/Mesh/Segmentation/ Combine Labels”. Estimate the width of the cell border for your sample in micrometers (for example, try 1 µm) and put it as the value for the “Border distance” parameter. The “Threshold”

parameter indicates the ratio of cell wall brightness to the brightness of cell interior. Therefore, it should be higher than 1. The exact value depends on the quality of signal in your sample. For the sample data, try a value between 1.5 and 1.8. Two cells will be merged if their ratio of common border signal vs. average signal is lower than the threshold value.

If you want to perform all the above processes as one, run "Process/Mesh/Segmentation/Auto-Segmentation". You will be able to set all the necessary parameters and decide whether or not you want to perform signal normalization.

- **Correct errors.** Examine the surface carefully and search for segmentation errors, especially in the regions where the signal is relatively low. Correct them in the same way as for manual segmentation.

Troubleshooting

- Symptom: "**Cells are under-segmented (not all cell walls are recognized)".** Try lowering the cell radius during signal blurring and seeding.
- Symptom: "**The mesh combine regions process creates a lot of errors (separate cells get merged for no reason)".** Try increasing the border distance or slightly lowering the threshold (remember not to go below 1). If the cells have signal inside (white spots inside the cells), try to normalize the mesh signal.

8. Refine the subdivision.

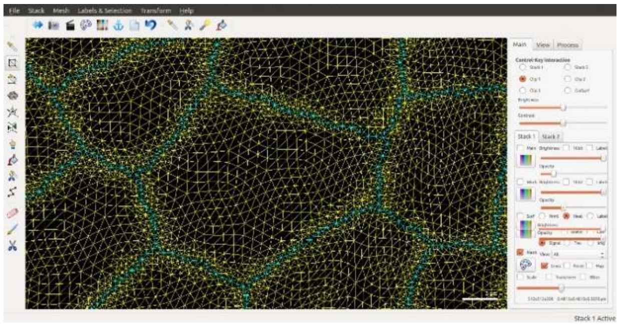

In order to minimize the number of vertices in the mesh, we will first do a coarse segmentation and then refine it by subdividing only near the cell boundaries. This is important especially if your computer is not very fast. Proceed as follows:

- **Subdivide near the walls.** Run "Process/Mesh/Structure/Subdivide Adaptive Near Border". This will clear the labels within a given distance from the border, and subdivide triangles larger than the max area specified in the parameters.

- **Re-project and re-segment.** Re-project the signal, smooth it, and then re-run the segmentation. This plus the previous operation can be repeated several times until the image is sharp. Sometimes it is necessary to change the parameters if the distance is too small, or the area too large. The idea is to get to the resolution of the stack. You will be able to tell when it is fine enough when you can start to see the voxels in the projected image when you zoom in. Now the image projected onto your mesh should look nice and sharp, and the cell borders should be much less jagged. If your computer is fast enough or your stack small you can subdivide the entire mesh ("Process/Mesh/Structure/Subdivide") before segmentation and omit this point.

9. Quantify Cell Area.

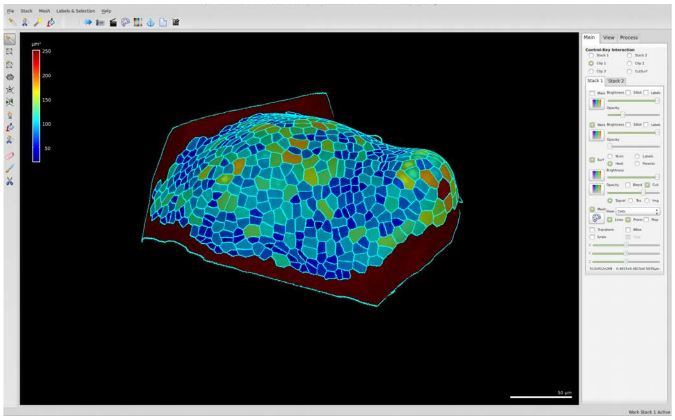

Now that the mesh is segmented into cells, you can do some analysis on it. To make a heat-map of cell area: Run "Process/Mesh/Heat Map/Heat Map". A dialog will pop up asking for parameters:

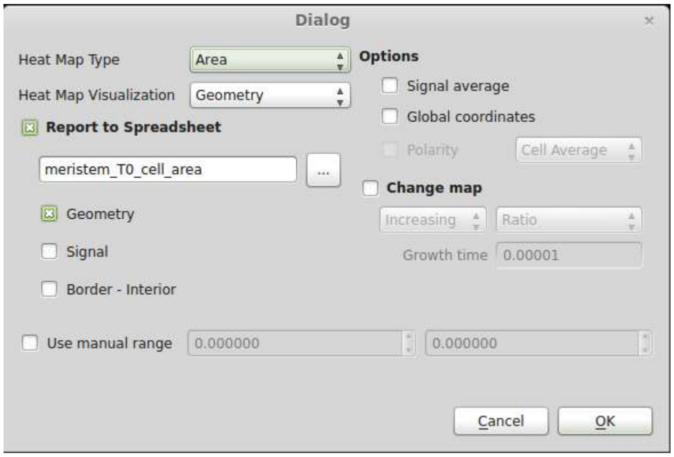

Be sure the heat map type is set to Area, and that the Report to Spreadsheet box is checked. Type in a filename for the spreadsheet. Hit OK and the cells will be colored by area. In case one of the labels covers a much greater area than the other, the automatic color map scaling will result in

most of the cells being blue and the large cell (e.g. border of the mesh) colored in red. Run “Process/Mesh/Heat Map/Rescale Heat Map” with proper parameters (in this example, Min = 20 and Max = 250) to re-adjust the color scaling.

The cell labels are listed along with their areas. If you click the Map checkbox inside MorphoGraphX, it will show the cell label numbers. You can also use the select the “Pick label” tool in the Mesh toolbar to find out which label belongs to which cell.

Label	Value	Area (µm²)	Center_X	Center_Y	Center_Z
11	48.8824	48.8824	50.4784	-75.0226	-15.8784
12	63.5403	63.5403	56.0198	-71.792	-16.3212
13	55.2761	55.2761	60.9629	-69.9763	-17.7705
14	77.1842	77.1842	66.1393	-64.5359	-14.7436
15	54.2675	54.2675	73.2261	-59.1079	-12.6078
16	[illegible]	[illegible]	[illegible]	[illegible]	[illegible]
17	54.1815	54.1815	78.292	-40.6395	-0.896271
18	35.7367	35.7367	82.4887	-34.1991	0.561473
19	48.8837	48.8837	82.2042	-16.8996	7.12617
22	44.182	44.182	83.6806	7.03876	8.70603
23	25.3023	25.3023	82.2381	2.04306	9.36707
24	37.9115	37.9115	85.3274	-4.73887	7.77237

Cell label

Value associated to color map = cell area

Cell center coordinates (x,y,z).

Example of spreadsheet output after running “Heat Map” of type “Area, Geometry”. This file is contained in the sample data folder under “meristem_T0_cell_area.csv”.

10. Quantify Fluorescence.

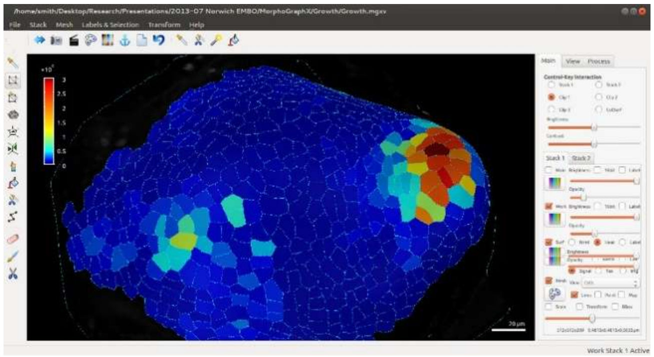

When imagining it is possible to collect fluorescence signal for gene expression on another channel. We can now analyze this data based on your segmentation:

- **Load the Data.** Use the Stack menu to load the fluorescence channel you want to quantify into the Main or Work store of the stack corresponding to your mesh. In this example, you can load “meristem T0 DR5.mgxs” into Stack1, Work.
- **Project the data onto the mesh**. Run “Process/Mesh/Signal/Project Signal”. At this point, be sure to have the surface visualization set to Normal, the Mesh visualization set

to Cells and the correct stack active (e.g. Work Stack1). You should now see which epidermal cells are expressing DR5 at a given depth. The default parameters Min Dist and Max Dist are set to 1 and 5, which means that the signal is taken only between 1 and 5 µm away from the curved surface. This depth corresponds to the epidermal layer in the example tomato meristem data. Try to change the depth parameter (e.g. 6 and 12, corresponding to the sub-epidermal layer) to see the effect on the projection, and compare this with the whole stack.

- **Make a heat map.** Run the same heat map as before, only this time set the heat map visualization to Total Signal in the dialog and check Signal for the spreadsheet. You now have a heat map of epidermal DR5 expression.

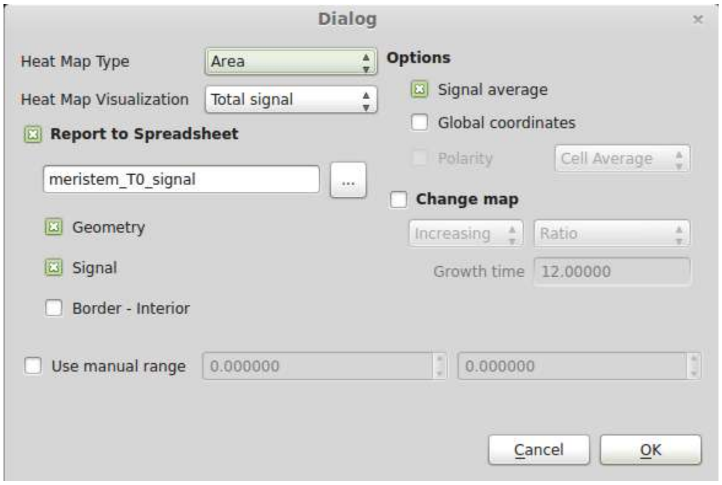

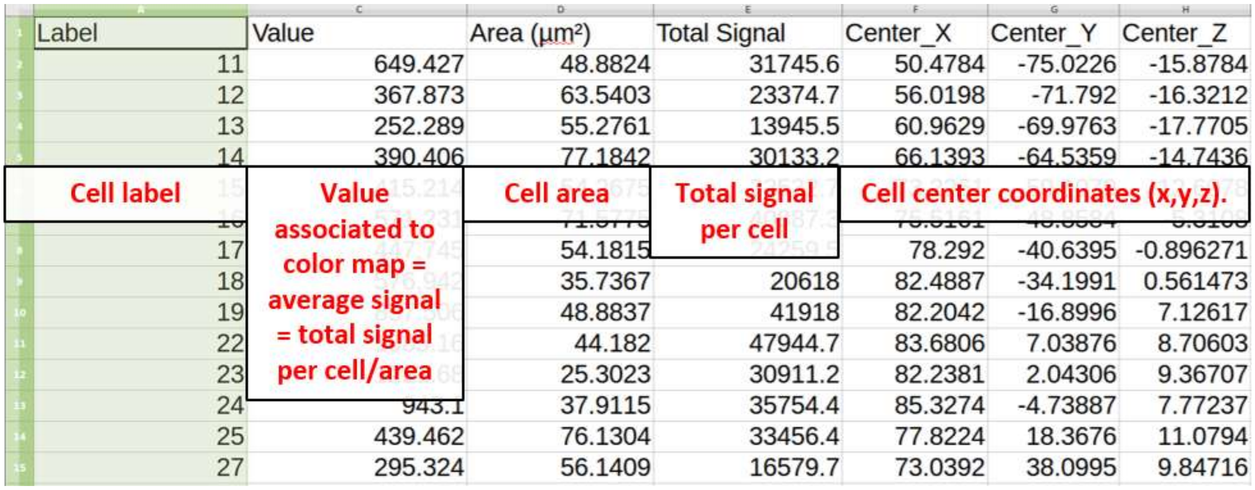

Label	Value	Area (µm²)	Total Signal	Center_X	Center_Y	Center_Z
11	649.427	48.8824	31745.6	50.4784	-75.0226	-15.8784
12	367.873	63.5403	23374.7	56.0198	-71.792	-16.3212
13	252.289	55.2761	13945.5	60.9629	-69.9763	-17.7705
14	390.406	77.1842	30133.2	66.1393	-64.5359	-14.7436
15	415.214	[illegible]	[illegible]	[illegible]	[illegible]	[illegible]
16	[illegible]	[illegible]	[illegible]	[illegible]	[illegible]	[illegible]
17	[illegible]	54.1815	[illegible]	78.292	-40.6395	-0.896271
18	[illegible]	35.7367	20618	82.4887	-34.1991	0.561473
19	[illegible]	48.8837	41918	82.2042	-16.8996	7.12617
22	[illegible]	44.182	47944.7	83.6806	7.03876	8.70603
23	[illegible]	25.3023	30911.2	82.2381	2.04306	9.36707
24	943.1	37.9115	35754.4	85.3274	-4.73887	7.77237
25	439.462	76.1304	33456.4	77.8224	18.3676	11.0794
27	295.324	56.1409	16579.7	73.0392	38.0995	9.84716

Example of spreadsheet output after running "Heat Map" of type "Area, Total signal", with "Signal average". This file is contained in the sample data folder under "meristem_T0_signal.csv".

11. Parent labeling.

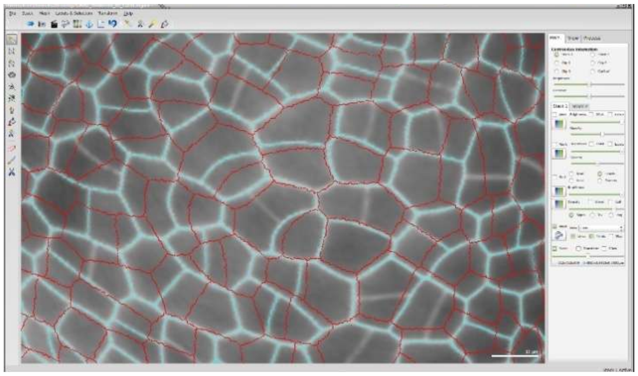

In order to make a growth map, we need to first segment a second time point of the time-lapse in the same way at the first one. In this example we assume that data for the first time point is stored in Stack1 and for the second one in Stack2. Of course labels on the first time point will not correspond to the second time point. In order to compute growth map we first need to associate the labels of the “daughter cells” (in Stack2) with the labels of their “parents” (in Stack1). MorphoGraphX provides a very simple way to do that:

- Go to **Stack1**. In the Main tab, turn “Stack” off. Turn “Surface” off with “**Labels**” active. Turn “Mesh” on with “View: Cells” option selected.
- Go to **Stack2**, Turn “Stack” off. Turn “Surface” on with “**Parents**” active. Turn “Mesh” on with “View: Cells” option selected. To easily distinguish the cell mesh of the second time point from the first one, change its color using the Colors Editor 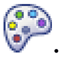.
- Now set the **Control-Key-Interaction to Stack1** on the “Main” tab. Hold the “Control” key and move the wireframe cell out line above Stack2. Move it to be about 2x the height of the sample above Stack2. Now rotate the view to look from the top. Looking from the top, try to align a few center cells as precisely as possible.If the growth was relatively large you can also scale Mesh1 to increase its size.
- **Transfer labels from Mesh1 to parents of Mesh2**. Make sure the “Surface” visualization and “Parents” is checked for Stack2, and that “Stack2” is active (i.e. Stack2 tab is selected). All editing operations work only on the active stack. Select the “Grab Label” 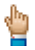 from the Mesh toolbar on the left. Now when you hold the Alt key, the tool will transfer the label of the cell you are looking through from Mesh1 onto parents of Mesh2. Try a few and verify that you are getting the correct labels. Transfer all of the labels in this way. In the second time point it may happen that some of the cells have divided. Both daughter cells will get the same label but will remain separated.
- **Save the parent labels.** Run “Process/Mesh/Lineage Tracking/Save parents”. NB: make sure that the stack of the second time point (in this case, Stack2) is active when you save the parents! Otherwise you will save an empty file.

Troubleshooting

- Symptom: **“It is not possible to superimpose all cells at the same time”.** This is normal. Start parent labeling with a few cells in the center of your sample and then move Stack1 along Stack2 while gradually adding new parent labels.
- Symptom: **“Samples in corresponding time points have very different sizes”.** In either Stack1 or Stack2, check the “Scale” box on the bottom of the stack tab. Now you can scale your stack along all three axes, which can make parent labeling easier.

12. Heat map of growth and cell proliferation.

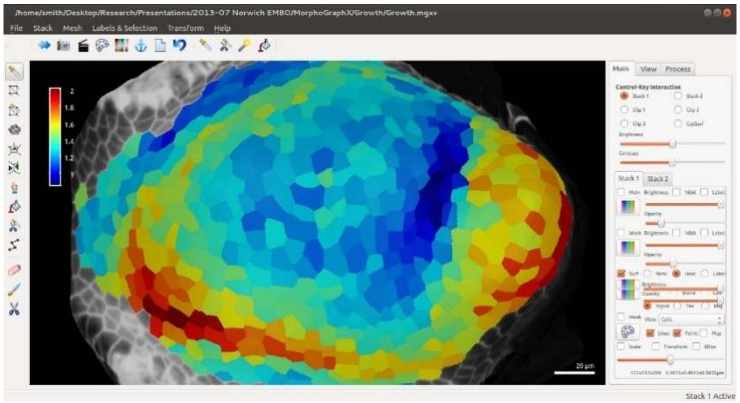

Now we are ready to make a map of cell growth and proliferation. This will correspond to the change in area of cells and number of cells over the time point. In order for this to work, it is important that the parent labeling (lineage tracking) is perfect. Mistakes will appear as outliers in the growth map and are very easy to spot. It is very unusual for lineage tracking to be perfect the first time.
To create the growth map:

- **Run the heat map process.** In Mesh1 select “Labels” and in Mesh2 select “Parents”. You should see corresponding cells on both meshes painted with the same color. Run “Process/Mesh/Heat Map/Heat Map”. Select “Area” for the heat map type and “Geometry” for the visualization. Also select the “Change map” box on. This tells MorphoGraphX to make a heat map comparing Stack1 and Stack2. The heat map can be visualized on either the first (typically, Stack1) or second (Stack2) time point. For the growth sample here, select “Increasing” if Stack 1 is active or “Decreasing” if Stack 2 is active. There are 3 options for the type of change map: “Ratio”, “Difference” or “Growth”. “Ratio” combined with “Increasing” will display the area of daughter cells (on stack2) divided by the area of their parents (on Stack1), so that a value of 1 means no growth and a value of 2 means 100% growth. “Difference” will give the area of daughter cells minus the area of their parents. The “Growth” option can be useful for comparing deformations occurring in series with different time intervals. The resulting values are

growth = (ratio of areas -1)/(time interval). If you want to export the results in a spreadsheet, check on the “Report to Spreadsheet” box.
NB: You can also quantify the change in signal between two time points. Run the “Heat map” process with the same parameters as for a growth map, except for “Heat Map Visualization”: “Total signal”. Use the “Signal average” option if you want to normalize the signal by cell size.

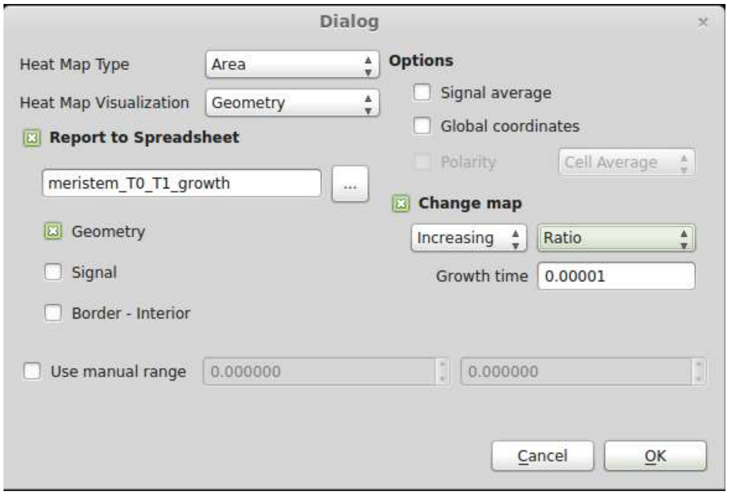

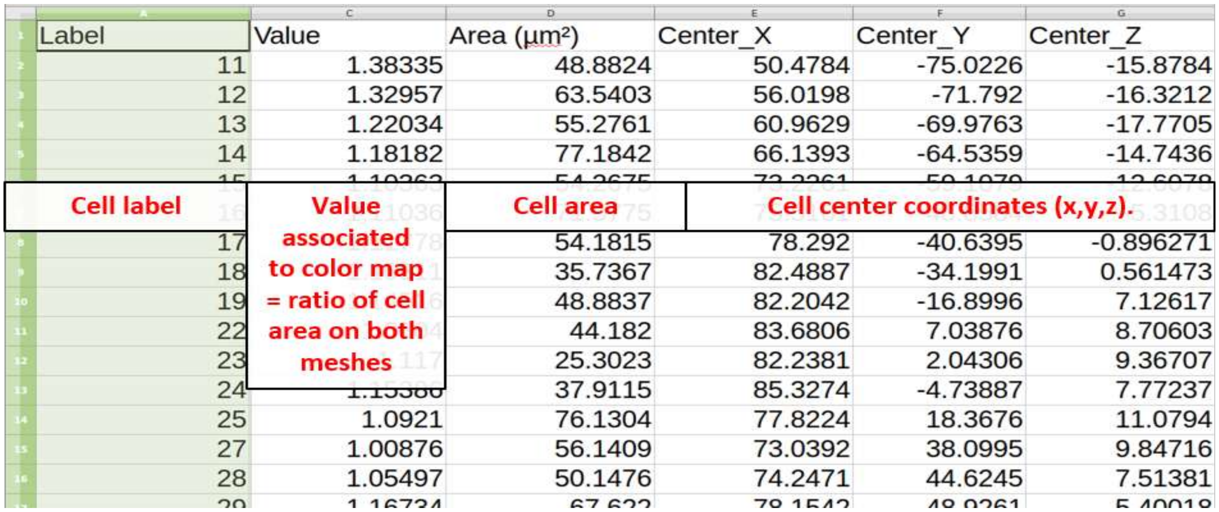

Label	Value	Area (µm²)	Center_X	Center_Y	Center_Z
11	1.38335	48.8824	50.4784	-75.0226	-15.8784
12	1.32957	63.5403	56.0198	-71.792	-16.3212
13	1.22034	55.2761	60.9629	-69.9763	-17.7705
14	1.18182	77.1842	66.1393	-64.5359	-14.7436
[illegible]	[illegible]	[illegible]	[illegible]	[illegible]	[illegible]
[illegible]	[illegible]	[illegible]	[illegible]	[illegible]	[illegible]
17	[illegible]	54.1815	78.292	-40.6395	-0.896271
18	[illegible]	35.7367	82.4887	-34.1991	0.561473
19	[illegible]	48.8837	82.2042	-16.8996	7.12617
22	[illegible]	44.182	83.6806	7.03876	8.70603
23	[illegible]	25.3023	82.2381	2.04306	9.36707
24	[illegible]	37.9115	85.3274	-4.73887	7.77237
25	1.0921	76.1304	77.8224	18.3676	11.0794
27	1.00876	56.1409	73.0392	38.0995	9.84716
28	1.05497	50.1476	74.2471	44.6245	7.51381
[illegible]	[illegible]	[illegible]	[illegible]	[illegible]	[illegible]

Example of spreadsheet output after running a heatmap with the parameters as shown above, on Mesh1. This file is contained in the sample data folder under “meristem_T0_T1_growth.csv”.

- **Fix problems.** If everything is perfect you will see a heat map like in the picture at the beginning of this section. More than likely you will see obvious outliers, individual cells which are much bluer than their neighbors, or much redder. Go to these cells and fix any segmentation errors. Note that the error might be in either Stack1 or Stack2, or it might have been a problem with the parent labeling transfer. Do not forget to save the parents after correction.

- **Create a cell proliferation map**. Run “Process/Mesh/Lineage Tracking”. Note that this process will work only for Stack2 where you have “Parents” active. This will display the heat map showing how many cells originate from one parent label.

13. Principal directions of growth (PDGs).

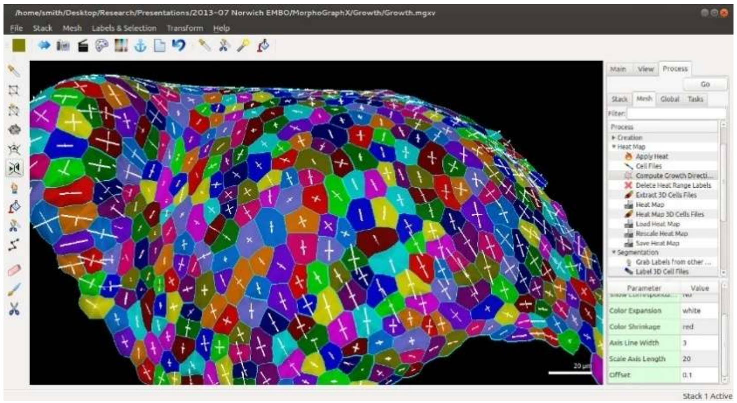

Principal directions of growth (PDGs) describe the growth anisotropy at the level of individual cells. The output of this process are vectors that, for each cell, give the direction of maximal and minimal growth and can be visualized as crosses. PDGs are computed based on cell junctions, i.e. the vertices that are at the intersection between 3 different labels. Before we can compute the PDGs we have to identify which junctions in the second time point correspond to junctions in the first one.

- **Fix corners.** Run “Process/Mesh/Cell Mesh/Fix Corners” for both meshes. This will make sure that all the junctions between cells are correctly segmented and that there is no triangle in the mesh that is left without a label. Fix Corners should report 0 vertices after running. Otherwise, run the process again. Save each mesh once Fix Corners is complete.
- **Find the correspondence between junctions**. Since the labels are different between the two meshes, load the parents in the second time point**.** Run “Process/Mesh/Cell Axis/PDG/Check Correspondence”. The output should be a color map, with cells which are correctly identified in blue and cells causing problems in red. The vertices from the first time point (Stack1) that could not be correctly identified in the second time point (Stack2) are selected and should appear in red. Make sure that “Mesh”, “Lines” and “Points” are selected for Stack1. At this point both meshes are simplified, so that only the vertices at the junctions between cells are present. *Do <u>not</u> over-write the meshes after running this process!*

  Correct the errors. If you compare the red cells on both meshes, you can identify the sources of errors in the correspondence between junctions. Most of the time they will be caused either by wrong parent labeling, which can be fixed easily by modifying the parent labels, or by an “exchange” in neighborhood (see illustrations below). In this case the original meshes will have to be modified. Open a separate MorphoGraphX session

with the original meshes. Keeping the first session open will help identifying which junctions should be corrected. Use the “Pick label” and “Add current seed” tools to modify the meshes. Save the meshes after correction and re-run “Check Correspondence” until all the cells are blue.

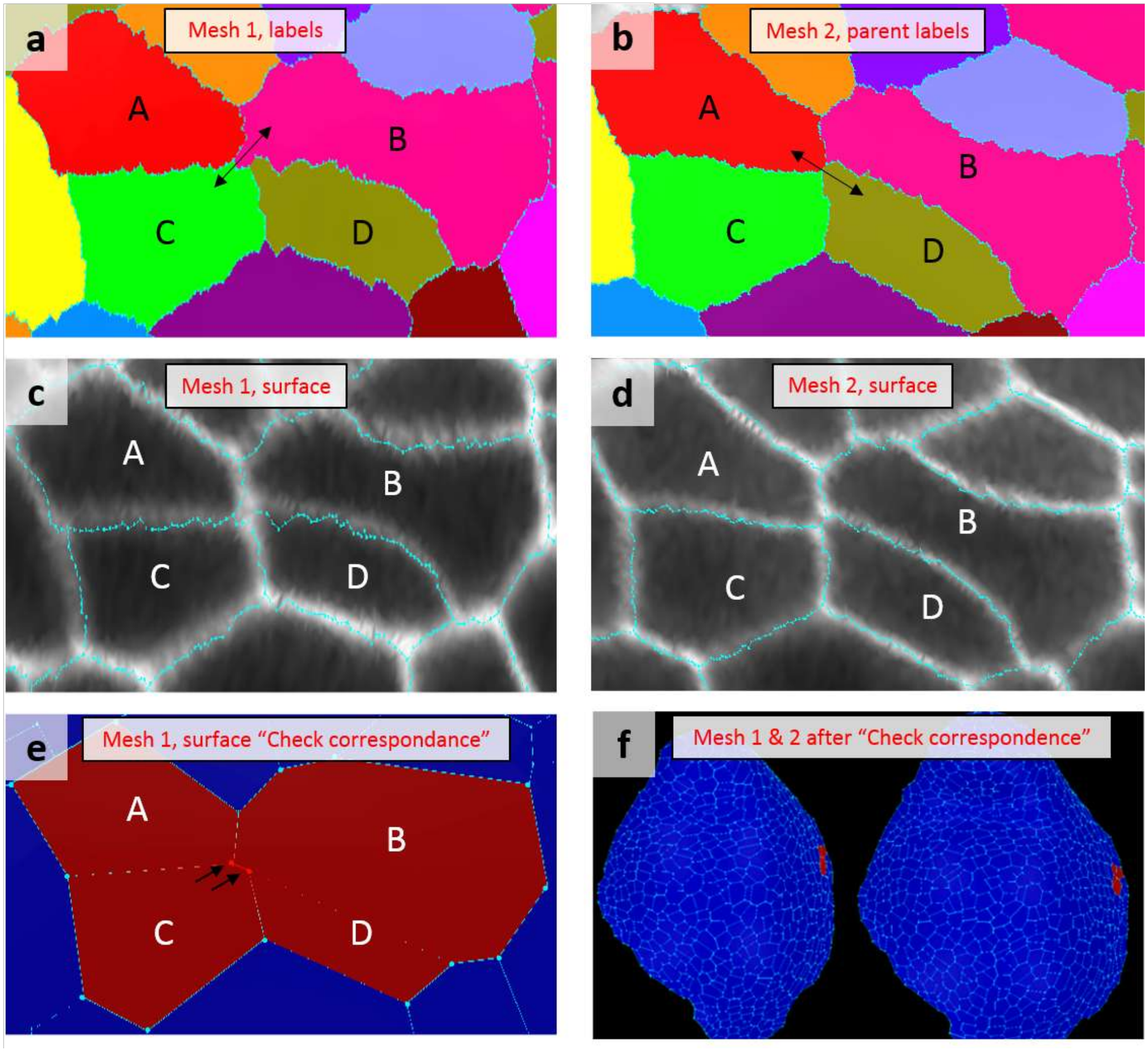

Typical segmentation error found by “Check Correspondence”. Junctions between cells are recognized in both meshes based on the identity of cells in contact. If 2 junctions are very close to each other it can happen that a small segmentation error lead to an “exchange” in neighbors. For example in Mesh1 (**a**) the cells B and C are in contact, forming 2 junctions (C,B,A) and (C,B,D). In Mesh2 (**b**), the cells A and D touch each other, forming the junctions (A,D,C) and (A,D,B), which do not exist in Mesh1. An inspection of the signal projection in Mesh1 (**c**) and Mesh2 (**d**) reveals that the segmentation is wrong on Mesh1, due to fuzzy signal in this region. The “neighbor exchange” is clearly made visible after running “Check correspondence”: cells involved are colored in red and the junctions of Mesh1 that could not be identified in Mesh2 are selected (arrows) (**e**). The faulty cells (in red) are easy to spot on the whole meshes (**f**).

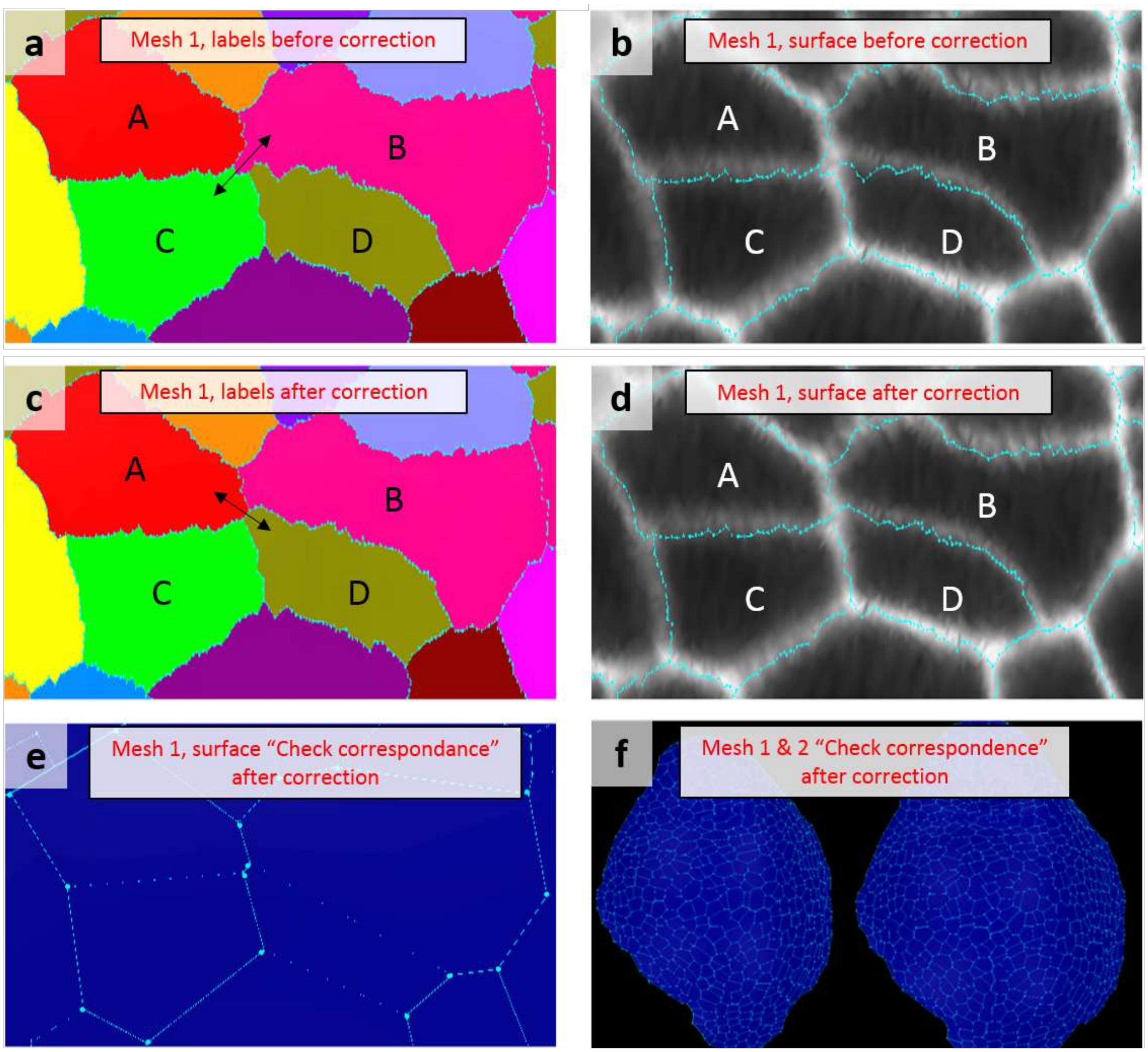

Correction of segmentation error. The "neighbor exchange" in Mesh1 (**a** and **b**) can be fixed (**c** and **d**) using the "Pick label" to select label "A" and "D" and "Add current seed" to re-label some of the mesh triangles. Remember: always save the mesh after correction! Re-run "Check correspondence" after correction. The cells should now appear in blue (**e**). If the entire meshes are free of mistakes (**f**), you can now proceed to the PDG computation.

- **Compute the PDGs.** Once the correspondence is complete, run "Process/Mesh/Cell Axis/PDG/Compute Growth Directions" with Stack1 active.
- **Save the PDGs** with "Process/Mesh/Cell Axis/Cell Axis Save". The maximal and minimal direction of growths (3D vectors) will be saved together with the values of deformation (stretch ratio) associated to them as a csv file (see image below).

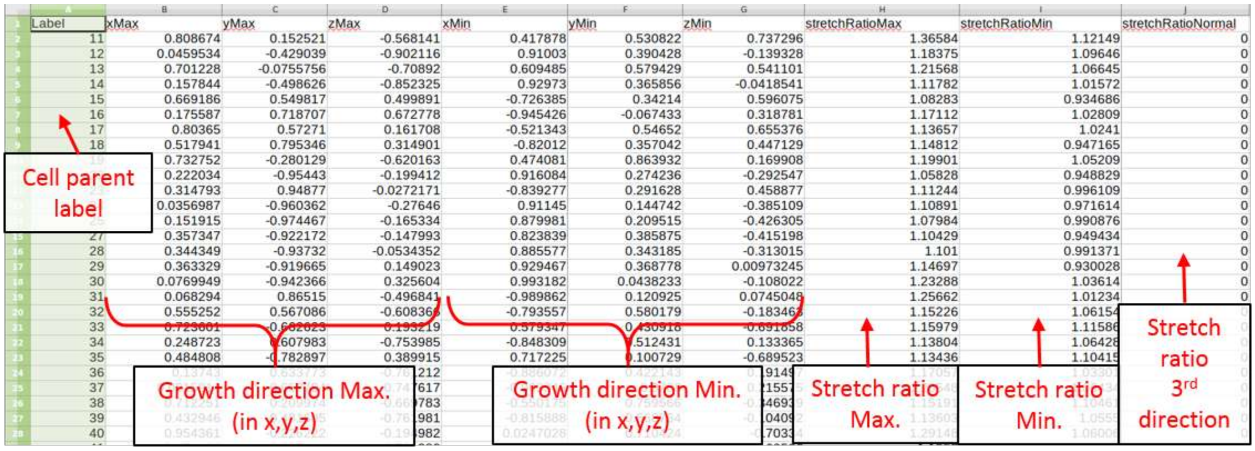

Label	xMax	yMax	zMax	xMin	yMin	zMin	stretchRatioMax	stretchRatioMin	stretchRatioNormal
11	0.808674	0.152521	-0.568141	0.417878	0.530822	0.737296	1.36584	1.12149	0
12	0.0459534	-0.429039	-0.902116	0.91003	0.390428	-0.139328	1.18375	1.09646	0
13	0.701228	-0.0755756	-0.70892	0.609485	0.579429	0.541101	1.21568	1.06645	0
14	0.157844	-0.498626	-0.852325	0.92973	0.365856	-0.0418541	1.11782	1.01572	0
15	0.669186	0.549817	0.499891	-0.726385	0.34214	0.596075	1.08283	0.934686	0
16	0.175587	0.718707	0.672778	-0.945426	-0.067433	0.318781	1.17112	1.02809	0
17	0.80365	0.57271	0.161708	-0.521343	0.54652	0.655376	1.13657	1.0241	0
18	0.517941	0.795346	0.314901	-0.82012	0.357042	0.447129	1.14812	0.947165	0
19	0.732752	-0.280129	-0.620163	0.474081	0.863932	0.169908	1.19901	1.05209	0
[illegible]	0.222034	-0.95443	-0.199412	0.916084	0.274236	-0.292547	1.05828	0.948829	0
[illegible]	0.314793	0.94877	-0.0272171	-0.839277	0.291628	0.458877	1.11244	0.996109	0
[illegible]	0.0356987	-0.960362	-0.27646	0.91145	0.144742	-0.385109	1.10891	0.971614	0
[illegible]	0.151915	-0.974467	-0.165334	0.879981	0.209515	-0.426305	1.07984	0.990876	0
[illegible]	0.357347	-0.922172	-0.147993	0.823839	0.385875	-0.415198	1.10429	0.949434	0
28	0.344349	-0.93732	-0.0534352	0.885577	0.343185	-0.313015	1.101	0.991371	0
29	0.363329	-0.919665	0.149023	0.929467	0.368778	0.00973245	1.14697	0.930028	0
30	0.0769949	-0.942366	0.325604	0.993182	0.0438233	-0.108022	1.23288	1.03614	0
31	0.068294	0.86515	-0.496841	-0.989862	0.120925	0.0745048	1.25662	1.01234	0
32	0.555252	0.567086	-0.608366	-0.793557	0.580179	-0.183463	1.15226	1.06154	0
33	0.723601	-0.682623	-0.193219	-0.579347	0.430918	-0.691858	1.15979	1.11586	0
34	0.248723	0.607983	-0.753985	-0.848309	0.512431	0.133365	1.13804	1.06428	0
35	0.484808	-0.782897	0.389915	0.717225	0.100729	-0.689523	1.13436	1.10415	0
36	[illegible]	[illegible]	[illegible]	[illegible]	[illegible]	[illegible]	[illegible]	[illegible]	[illegible]
37	[illegible]	[illegible]	[illegible]	[illegible]	[illegible]	[illegible]	[illegible]	[illegible]	[illegible]
38	[illegible]	[illegible]	[illegible]	[illegible]	[illegible]	[illegible]	[illegible]	[illegible]	[illegible]
39	[illegible]	[illegible]	[illegible]	[illegible]	[illegible]	[illegible]	[illegible]	[illegible]	[illegible]
40	[illegible]	[illegible]	[illegible]	[illegible]	[illegible]	[illegible]	[illegible]	[illegible]	[illegible]

Example of PDG output file from the "Cell Axis Save" process. The first column of the csv file gives the label of the cell on the first time point (which is the same as the parent label on the second time point). The columns "xMax", "yMax" and "zMax" give the (x, y, z) coordinates of the direction (unitary vector) of maximal growth, while the column "stretchRatioMax" contains the magnitude of stretch in this direction. Similarly, "xMin", "yMin" and "zMin" define the direction of minimal growth, "stretchRatioMin" the stretch ratio. "stretchRatioNormal" gives the value corresponding to the axis perpendicular to both minimal and maximal growth directions. Since we compute only the 2D deformation of the cell in its average plane, this third value is zero.

- **Display the PDGs.** You can now re-load the original meshes and display the PDGs on them. Make sure that parent labels are also loaded on the second time point before loading the PDGs. Load the PDGs with "Process/Mesh/Cell Axis/Cell Axis Load", "Type: PDG". In "Process/Mesh/Cell Axis/PDG/Display Growth Directions" you can choose what type of heat map to display. StretchMax (resp. StretchMin) is the value of deformation (stretch ratio) in the maximal (resp. minimal) direction. A stretch ratio of 1 means no deformation, 2 means an elongation by 100%, 0.8 a shrinkage of 20%. The product of StretchMax and StretchMin is equivalent to the areal growth of the cell polygon. The color and size of the PDG vectors can also be modified. By default, vectors corresponding to expansion (stretch ratio > 1) are displayed in white, while red is used to draw the direction of shrinkage (stretch ratio < 1). The "Threshold" parameter is used to display PDGs axis only in cells for which the anisotropy is above a given value.

14. 3D segmentation.

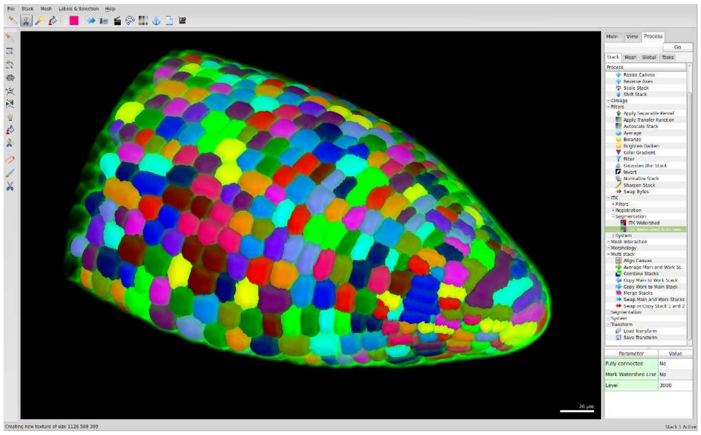

It is also possible to fully segment cells in 3D, with sufficient quality confocal stacks. Best results can be achieved with very dense stacks (fine Z step) and a small pinhole diameter. Since it is particularly difficult to seed in 3D, MorphoGraphX uses an auto-seeded watershed from the C++ library called the Insight Toolkit (ITK).

To do the 3D segmentation:

- **Load the stack.** You can use the example data (radicle of a mature Arabidopsis embryo) from the “Bassel_2014” folder provided on www.MorphoGraphX.org.
- **Blur to reduce noise.** Run “Process/Stack/Filters/Gaussian Blur”. Use a radius slightly larger than the width of the cell walls in the sample. In the example, try 1 µm.
- **Segment.** Run “Process/Stack/ITK/Segmentation/ITK Watershed Auto Seeded”. This can take some time, but you do not have to do any seeding. Adjust the threshold according to the results of the segmentation (here, try 3000). If a sample is highly under segmented (cells fused), then decreasing the threshold is needed. If the sample is over segmented (single cells segmented into multiple pieces), then the threshold needs to be increased.
- **Delete the outside.** The segmentation fills the entire volume with labels and the sample will be buried inside somewhere. To remove the outside label, select the “Delete Picked Label in Volume” 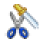 from the Volume tool bar. Be careful what you delete, as you cannot go back. You can, however, save the segmented volume (in the Work store) under the Stack menu.
- **Correct the segmentation.** Auto-seeded segmentation almost always requires correction for over segmented cells. These multiple segments can be fused together into a single segment by using the color picker 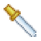 and bucket 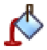 from the Volume tool bar to merge cells. Use the clipping planes under “View” tab (see section 3a) to correct for errors inside the sample.
- **Extract the volumes.** When you are happy with the segmentation, extract the mesh using “Process/Mesh/Creation/Marching Cubes 3D”. The “Cube spacing“ parameter defines how far apart vertices are in the mesh. For a fine and detailed mesh, cube spacing should be smaller (try 3 µm and above). The tradeoff to this is a larger file size for the mesh.

Cells are automatically labeled with the same values as the stack labels. Once the mesh is extracted you can edit each cell individually using the “Select Connected Area” 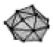 tool, which selects all vertices that have the same label.

- **Make a heat-map.** You can now make a heat-map to visualize the cell volumes. Run “Process/Mesh/Heat Map/Heat Map”. Select Volume for the Heat Map Type to color the cells by volume. It is also possible to make a heat map of area, or to count fluorescence inside volumes if you have that collected on a separate channel. Like the Area heat-maps, it is also possible to quantify cell expansion.

Note: To get an optimal 3D segmentation, experiment with different values for the blurring and the threshold parameter for the ITK segmentation to see if you can nicely segment all the cells with as little manual correction as possible. You cannot split cells, so if the stack is under segmented, then you must re-run the segmentation with a lower threshold. If a stack is highly over-segmented, then extensive manual correction is needed. Optimizing the segmentation in the first instance is the quickest way forward.

Troubleshooting

- Symptom: **“Error message after running ITK Watershed (‘*Number of objects greater than maximum of output pixel type.’*)”.** Increase the threshold to get less cells.
- Symptom: **“A lot of cells in the segmented stack have the same color”.** Make sure that the cells are not fused (have different labels) using the “Pick label from a volume” tool. If you have a lot of cells your samples (more than 30), check on the “16bit” option on the Work stack. This will change the label color display. Note that labeled stacks use a different color mapping from normal ones. The numbers representing the voxels are mapped to a color table which is typically 16 colors, and repeats. If the number of labels exceed 16, cells with different labels can be displayed with the same colors.
- Symptom: “**The software is slow to run after mesh extraction**”. Adjust the cube size for the 3D marching cubes to see the effect on the shape extraction of the cells. Do not go too small or it will create too many vertices and be very slow to run. If the cube size is too large then the shapes of the cells will not be accurately captured.

15. Create your own workflow (tasks).

While processing data you will often use the same processes in a particular order. It is then convenient to organize the processes into tasks.

To create and edit tasks, right click below the Tasks tab. Click on “New task” in the Edit User Task window. Select the processes and drag them in the right panel.
To save the tasks, you can export them into a text file. Saving the MorphoGraphX session file (.mgxv format) will also save the parameters used in the tasks.

Reference

16. Installation

MorphoGraphX is straightforward to install, however it can be tricky to get the nVidia graphics drivers and Cuda installed correctly. Install the newest driver for your graphics card and the newest version of Cuda that is available in the repository for your distribution. If you have problems, it may be necessary to install a newer version from the nVidia web site.

MorphoGraphX is available as a Debian package which can be downloaded from www.MorphoGraphX.org. Binary versions are available for Linux Mint and Ubuntu. It is important to get the correct release, as different versions of Linux ship with different versions of the libraries that MorphoGraphX depends on. This is particularly true for the Cuda GPU tools, which are evolving rapidly.

Use your favorite software manager to install the MorphoGraphX package (or just double click on it). In order to use the 3D segmentation and other tools from the Insight Toolkit, you will need to install the ITK package as well. A compiled version of the ITK package is available on the MorphoGraphX web site for convenience, but does not differ from the version available from www.itk.org. If you would prefer to compile ITK yourself, be sure to enable the Module_ITKReview in CMake to use the MorphologicalWatershed filter.

After installation – enabling the ALT key

After MorphoGraphX has been installed, it should show up in the menu system, or you can start it by typing "mgx" at the command line or double-clicking on a MorphoGraphX project (.mgxv), mesh (.mgxm) or stack (.mgxs) file. When MorphoGraphX first starts, double check that there are no errors in the terminal window. If the graphics card is identified properly, and the is memory allocated to Cuda, it is most likely everything install correctly.

The next task is to enable the ALT key. In Linux Mint and Ubuntu, the ALT key is used to move windows around. MorphoGraphX uses this key for user interaction, so you will need to change the key used by Linux to the "Super" (or Window) key. In Mint cinnamon, this is done by right clicking on tasks bar, and going into "All-Settings" - "Windows". There you change the mouse modifier key from Alt to the Super key.

Laptop use

MorphoGraphX will run on a laptop that has a dedicated nVidia graphics card. Most newer laptops will use nVidia Optimus technology. In this case the laptop will use the graphics card built into the processor for simple tasks, and will only turn on the dedicated card when required. This will be handled automatically by the MorphGraphX startup script, but it is necessary to get the Bumblebee and/or Primus software working on your machine.

Troubleshooting

- Symptom: The viewer window is not black, you get shader errors in the terminal window, or when you load a stack it is a single solid color. This means that the nVidia graphics driver is not installed properly.

- Symptom: Libraries (.so files) not found. Sometimes libraries are not found immediately after installation. At the command line type:

  ```
  $ sudo ldconfig
  ```

- Symptom: You see the following error message in the terminal window when MorphoGraphX starts: "Cuda holdMem, cannot allocate 8 Meg, giving up"This means that the Cuda driver is not installed properly. If you can get any application that uses Cuda working, then MorphoGraphX should work as well

Note: There appears to be a problem with the Cuda driver in the Ubuntu 14.04 repository. To see if this problem affects you, try starting MorphoGraphX as root at the command line:

```
$ sudo mgx
```

If this solves the problem, MorphoGraphX will work without root privilege until the system is rebooted. The issue is a problem with the Cuda library not creating the device files properly, it has been reported here:

https://devtalk.nvidia.com/default/topic/699610/linux/334-21-driver-returns-999-on-cuinit-cuda-/

This can be fixed by editing the file: "/etc/rc.local" and add the following lines to it:

```
modprobe nvidia-uvm
mknod -m 666 /dev/nvidia-uvm c $(grep nvidia-uvm /proc/devices | cut -d \  -f 1) 0
chgrp video /dev/nvidia-uvm
```

This will create the necessary device files at startup.

Cuda 6.5

An alternative option is to install Cuda 6.5, which is the nVidia officially supported version for Ubuntu 14.04. If you go this route, you will need to download a version of MorphoGraphX with Cuda6.5 in the package name. Also you will need to put the following lines in your .bashrc file:

```
export CUDA_HOME=/usr/local/cuda-6.5
export LD_LIBRARY_PATH=$LD_LIBRARY_PATH:${CUDA_HOME}/lib64
export PATH=${CUDA_HOME}/bin:${PATH}export
```

17. Writing custom processes (plugins)

It is possible to extend MorphoGraphX by writing your own processes. Processes can be compiled into shared object (.so) files and are loaded when MorphoGraphX starts. They can be installed in a system area for all users, or in the user's home directory. Run the command:

```
$ MorphoGraphX --all-process
```

to print the plug-in directories. An overview for the process documentation can be found in the “process” namespace in the Doxygen programmer documentation, which is available from the help menu in MorphoGraphX. After installing MorphoGraphX, this documentation can also be found here: “/usr/local/share/doc/MorphoGraphX/html/index.html”.

The best way to start developing processes is to start from a sample available from the MorphoGraphX website: www.MorphoGraphX.org. In the “Samples” plug-in pack there are the following simple processes:

StackGammaFilter - This process implements a simple gamma filter on the stack. It demonstrates how to read and write information from the stack, and how to collect parameters from the user interface. Note that this process inherits from the StackProcess class.

MeshGammaFilter - This process implements a simple gamma filter on the mesh signal. In order to use this process, you will need to have extracted a mesh, and projected the signal on in (see Section 6). Note that this process inherits from the MeshProcess class.

ExportMeshToFile - This process demonstrates how to write mesh data to a text file. It sames the mesh in simple OBJ (Wavefront) and PLY (Stanford Polygon) formats. The process also demonstrates how to use drop-down pick boxes in the GUI and how to call a dialog for file selection.

ITKMedianImageFilter - This plugin demonstrates how to cell filters from the Insight Toolkit (ITK) image processing library. MorphoGraphX provides an image “source” for input, and a “sink” that writes the data back to the work store.

To compile a plugin, extract the archive into a directory. You can then type:

```
$ qmake
$ make
$ sudo make install
```

By default, the processes are set to install into the system area. In cases where you do not have sudo rights, or you wish to install only for yourself, you can copy the “.so” file to your local process directory. This will be in your home directory under:

“.local/share/data/MorphoGraphX/MorphoGraphX/processes”. See above to list the process directories.

To create a new process, it is easiest to copy an exisiting one. For the StackGammaFilter process, there are 5 files:

StackGammaFilter.hpp – C++ process headerfile
StackGammaFilter.cpp – C++ process file
StackGammaFilter.png – Icon file
StackGammaFilter.qrc – Qt resource file (points to icon file)
StackGammaFilter.pro – Qmake project definition file.

In order to compile processes you will need to have g++ and the Qt4 development tools installed.

18. Compiling MorphoGraphX from source

Linux

The source code for MorphoGraphX is released alongside the binary distributions. CMake is used for the build system. In order to compile MorphoGraphX, you will need to install the following packages:

g++
libqt4-dev
libqt4-opengl-dev
libglew-dev
cimg-dev
libgsl0-dev
libtiff5-dev
cmake-gui
python-dev
doxygen

Windows

Currently, only Linux is supported, although previously versions have been compiled for Windows. To compile on Windows, you will need g++ (for example with MinGW), however Cuda requires MSVC. To handle this, the Cuda code is compiled to a separate .dll. It is pretty straightforward to set up MinGW and MS Visual Studio Express (free) for 32bit, however this gets much more complicated if you would like to compile a 64 bit version.

Mac

The OpenGL support on Mac (too poor/always changing) means that it is unlikely to be possible to compile on Mac.

Neither Windows nor Mac have been tested, so there are likely to be many bugs.

19. File formats

MorphoGraphX can read and write different formats for both mesh and stack files. A summary of the formats available is a follows:

Stack		
.mgxs	read/write	MorphoGraphX custom stack file format. See the StackSave process in the Doxygen for a detailed description of the format.
.inr	read/write	Format developed at INRIA, Montpellier
.tiff	read/write	Compatible with multipage tiff files written by ImageJ
Stack Import		
ITK	read	Stack formats readable by ITK (Insight Toolkit)
image series	read	Series of 2D images. Any format readable by CImg. If ImageMagik is installed, Cimg can convert from almost any format.
Mesh		
.mgxv	read/write	MorphoGraphX custom mesh format. See the MeshSave process in the Doxygen for a detailed description of the format.
.ply	read/write	Stanford polygon file format.
.vtk	read/write	VTK (Visualization Toolkit) format.
.mesh	read/write	MeshEdit format.
.stl	write	StereoLithography CAD file format.
.obj	read/write	Wavefront OBJ format.
.txt	read/write	Custom simple text format. See the MeshExport process in the Doxygen for a detailed description of the format.

20. Command line options

Configuration information is available by launching MorphoGraphX from the command line. To view all the available options:

```
$ MorphoGraphX --help
```

```
Usage: MorphoGraphX [--debug|--dir|--process|--all-process|--help|-h] [FILE.mgxv]

   --debug           - Launch MorphoGraphX in debug mode
   --dir             - Print the application directory and exit
   --process         - Print the process directory and exit
   --user-process    - Print the user process directory and exit
   --all-process     - Print all the directories searched for processes and exit
   --include         - Print the include directory and exit
   --version         - Display the version and revision and exit
   --help |-h        - Print this help
```
